# Carcinoma of Unknown Primary (CUP) versus CUP Turned to Primary Carcinoma of the Head and Neck—An Analysis of Diagnostic Methods and the Impact of Primary Tumor on Clinical Outcome

**DOI:** 10.3390/diagnostics12040894

**Published:** 2022-04-03

**Authors:** Muhammad Faisal, Nguyen-Son Le, Stefan Grasl, Stefan Janik, Helmut Simmel, Annemarie U. Schratter-Sehn, Jafar-Sasan Hamzavi, Peter Franz, Boban M. Erovic

**Affiliations:** 1 Department of Surgical Oncology, Shaukat Khanum Memorial Cancer Hospital and Research Center, Lahore 54000, Pakistan; maxfas@live.com; 2 Institute of Head and Neck Diseases, Evangelical Hospital, 1180 Vienna, Austria; sasanhamzavi@me.com; 3 Department of Otorhinolaryngology, Head and Neck Surgery, Medical University of Vienna, 1090 Vienna, Austria; sonle1993@hotmail.com (N.-S.L.); stefan.grasl@meduniwien.ac.at (S.G.); stefan.janik@meduniwien.ac.at (S.J.); 4 Institute of Radio-Oncology, Kaiser-Franz-Josef Hospital, 1100 Vienna, Austria; helmut.simmel@wienkav.at (H.S.); annemarie.schratter@wienkav.at (A.U.S.-S.); 5 Department of Otorhinolaryngology, Head and Neck Surgery, Rudolfstiftung Teaching Hospital, 1030 Vienna, Austria; peter.franz@wienkav.at

**Keywords:** carcinoma of unknown primary, head and neck, true CUP vs. CUP turned to primary carcinoma, diagnostic methods, prognostic factors, outcome

## Abstract

Background. The purpose of this study was to analyze the value of different diagnostic methods in detecting the primary site and the impact of primary tumors on the clinical outcome of carcinoma of unknown primary (CUP). Methods. In this multicenter, retrospective study, 124 patients with true CUP (n = 94) and CUP turned to primary carcinoma (n = 30) were included. Patients with evidence of primary site during the clinical examination were excluded a priori. The diagnostic procedure was comprised of imaging and invasive methods (fine-needle-aspiration, tonsillectomy and panendoscopy). All patients were treated with curative intent. Results. Despite extensive diagnostic workup, the primary site remained unknown in 75.8%. Invasive diagnostic methods showed higher primary detection rates than imaging modalities (15.1% vs. 7.8%). Tonsillectomy and panendoscopy revealed the primary tumor in 14.9% and 15.2% of patients, whereas the detection rates of CT, MRI and FDG-PET-CT were 10.1%, 4.8% and 6.5%, respectively. The occurrence of primary tumors led to a significantly deteriorating 5-year overall survival (*p* = 0.002) and emerged as survival prognosticator (HR = 2.764, *p* = 0.003). Conclusion. Clinical examination in combination with tonsillectomy and panendoscopy was superior to imaging alone in detecting the primary tumor. When the CUP of patients turned to a primary tumor, clinical outcome was significantly worse than in CUP patients.

## 1. Introduction

Carcinoma of unknown primary (CUP) is a rare disease entity that is defined as lymph node metastases without identification of a primary site despite comprehensive diagnostic workup [1]. The incidence of cervical lymph node metastasis (LN), derived from an unknown primary tumor, ranges from 2–10% of all head and neck tumors [2,3,4]. Theories indicate inter alia, subclinical dormancy, anatomic complexity of the head and neck, microtumors and spontaneous tumor involution as speculative explanations for difficulties in detecting the primary site [1,5,6]. Despite the extensive literature, a standard diagnostic algorithm is still the subject of recent research [7]. However, a diagnostic algorithm generally comprises patients’ anamnesis, clinical examination, imaging modalities including ultrasound, computed tomography (CT), magnetic resonance imaging (MRI) and/or fluorodeoxyglucose positron emission tomography-computed tomography (FDG-PET-CT), fine needle aspiration cytology (FNAC) or ultrasound-guided core biopsy, panendoscopy and tonsillectomy [7]. The importance of comprehensive diagnostics is explained by the indicative guidance of the histology and the affected neck level of the LN in the primary tumor search [1,8]. Mostly, cervical metastases involve levels II and III [1,3,7,8] and appear as squamous cell carcinomas (65–80%), followed by undifferentiated carcinomas (10–14%) and adenocarcinomas (13%) [2,8,9]. In the literature, the imaging methods seem to reach the limits as detection rates of 22% for CT, 36% for MRI and 25–37% for PET-CT are described [6,10,11]. As part of the invasive diagnostic procedure, tonsillectomy and panendoscopy lead to primary identification rates of 11–23% [4,9] and 31% [9], respectively.

The importance of detecting the primary site is discussed controversially as the prognostic impact could not be shown sustainably but, however, definitely allow more tumor-specific and less aggressive therapy [12,13,14].

The primary aim of the study was to analyze the value of diagnostic methods regarding the identification of primary tumors in CUP of the head and neck and to help clinicians in directing future diagnoses. Furthermore, we hypothesized that the occurrence of the primary site is a substantially deteriorating prognosticator regarding the clinical outcome, and, therefore, we compared true CUP with CUP turned to primary carcinoma (tCUP).

## 2. Materials and Methods

### 2.1. Patient Cohort

In this multicenter, retrospective study, the data of 124 patients with true CUP (group A) and CUP turned to primary carcinoma (tCUP—group B) of the head and neck were assessed, respectively. True CUP was defined as cervical LN with occult primary tumor despite extensive diagnostic examinations. In patients with CUP turned to primary carcinoma, the clinical examination, including flexible nasopharyngoscopy remained perfectly inconspicuous regarding possible evidence of the primary tumor, which, however, occurred later during imaging or invasive diagnostics.

From 1993 onwards, the patients have been treated at the (I) Departments of Otorhinolaryngology, Head and Neck Surgery of the Vienna General Hospital (University Hospital of the Medical University of Vienna), (II) Hanusch Hospital (Vienna), (III) Kaiser-Franz-Josef Hospital (Vienna) and (IV) Rudolfstiftung Teaching Hospital (Vienna). In order to homogenize the patient cohort, patients had to fulfill our inclusion and exclusion criteria. All patients with cervical lymph node metastasis and inconspicuous clinical examination were included. Accordingly, patients with even the smallest clinical suspicion of a primary tumor were excluded. Patients with occurred primary tumors during the follow-up were placed in group A. Hence, 94 patients of group A and 30 patients of group B could be included in the diagnostic analyses, respectively.

The emergence of distant metastases at the time of diagnosis and patients with palliative therapy protocol or prematurely terminated therapy (n = 18) were subject to our exclusion criteria for the outcome analyses.

The study protocol was approved by the Ethics Committee of the Medical University of Vienna (ethics commission number: 2303/2016).

### 2.2. Diagnostic Methods

The standard diagnostic algorithm comprises three parts: (I) clinical examination, (II) imaging modalities and (III) the invasive diagnostic part. First, a detailed patient anamnesis and medical history were obtained, followed by a thorough clinical examination, including flexible nasopharyngoscopy. Subsequently, ultrasound of the neck, FNAC or ultrasound-guided core biopsy, chest X-ray, CT/MRI and/or 18F-FDG-PET-CT were performed. Panendoscopy and tonsillectomy were carried out as invasive diagnostic procedures.

### 2.3. Treatment Methods

According to the institutional therapy protocol of group A, lymph node extirpation and/or neck dissection (ND) was performed initially. Depending on the TNM stage, unilateral or bilateral ND was applied. Following the surgical interventions, all patients received either unilateral or bilateral radiotherapy using three-dimensional conformal radiation therapy (3D-CRT), intensity-modulated radiation therapy (IMRT) or volumetric modulated arc therapy (VMAT) with doses of 66 to 70 Gy, 2–2.2 Gy per fraction, on weekdays over approximately 6 to 7 weeks. For lower-risk anatomical areas, lower doses were used between 43 and 66 Gy. Furthermore, chemotherapy (CTX) was applied in certain patients, such as extranodal extension in final histology or residual disease not amenable to resection and HPV-related cancers requiring altered chemoradiation protocols.

Depending on the localization, tumor resection, RT and/or CTX were additionally performed as a primary tumor treatment in group B.

### 2.4. Statistical Methods

**Figure 1:**
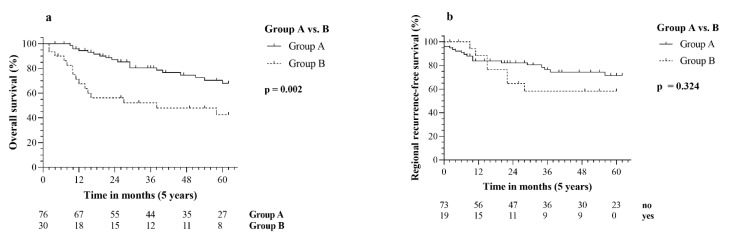
Kaplan–Meier curves: (**a**) 5-year overall survival and comparison of group A (CUP) vs. group B (tCUP); (**b**) 5-year regional recurrence-free survival and comparison of group A (CUP) vs. group B (tCUP).

SPSS^®^ version 24 (IBM, Armonk, NY, USA) was used for the statistical analyses. By performing Kaplan–Meier survival analysis, the 5-year overall survival (OS) and 5-year regional recurrence-free survival (RRFS) were estimated, and corresponding *p*-values were obtained via log-rank test. The Chi-square test was used to analyze the impact of the primary tumor on the emergence of distant metastasis. In order to analyze potential prognostic factors, univariate and multivariate cox regression analyses were performed by using Cox proportional hazards model. Variables with *p* ≤ 0.05 in the univariate tests were included in the multivariate analysis. Kaplan–Meier curves (Figure 1) were created in GraphPad Prism 8 (GraphPad Software, San Diego, CA, USA).

## 3. Results

### 3.1. Patient Characteristics

Within our cohort, the median age of the patients was 61 years (range: 38–88) with a male to female ratio of 3.8 to 1. At the time of the first presentation, nicotine consumption was stated in 56.5% of patients with an average of 24.9 cigarettes per day, while frequent or excess alcohol consumption was documented in 71.8% of patients. The family anamnesis was positive for cancer in 35.5% of patients (Table 1).

### 3.2. Imaging and Invasive Diagnostic Methods

In order to detect the primary tumor and to determine the extent of the disease, CT and/or MRI of the head and neck were performed in 82.5% and 34.4% of patients, while CT of the thorax and abdomen was part of the staging in 69.2% and 59.8% of cases, respectively. Furthermore, half of the patient cohort received a whole-body FDG-PET-CT scan.

In 35.5% of patients, FNAC was performed and led to a true positive diagnosis of LN in 59.1%. The application of panendoscopy was documented in 90.3% of cases. Tonsillectomy was carried out in 37.9% of patients, while the tonsils were already removed in 37.9% (Table 1).

### 3.3. Cervical Lymph Node Metastases

By classifying LNs according to the staging system of the AJCC [15], 13.7% of patients were staged as N1, 12.9% as N2a, 44.4% as N2b, 7.3% as N2c and 19.4% as N3. Level II and III emerged as the most commonly affected areas of the neck (level II: 44.6%, level III: 23.7%, cumulatively: 68.3%). Histopathological analysis revealed a squamous cell carcinoma (SCC) (89.5%) with moderate to poor differentiation (G2-G3, cumulatively: 81.5%) in the majority of cases, whereas adenocarcinoma (4.8%), undifferentiated carcinoma (1.6%) and neuroendocrine carcinoma (0.8%) were diagnosed rarely. p16 overexpression was observed in 54.3% of the documented cases (Table 2).

### 3.4. Primary Tumor

During the initial diagnostic workup, the primary tumor remained occult in 94 patients (group A—CUP, 75.8%) and was detected in 30 patients (group B—tCUP, 24.2%). Within group B, tonsillectomy turned out to have the highest primary tumor detection rate of 70.0%, followed by panendoscopy (60.7%). In 37.0%, 28.6% and 40.0% of cases, CT, MRI and FDG-PET-CT showed a high suspicion of the primary tumor, respectively, and were mostly confirmed by panendoscopy, CT-guided puncture, parotidectomy, biopsy in local anesthesia or tonsillectomy. The primary tumor was most commonly hidden in the tonsils (26.7%) and tongue base (23.3%), while it occurred in other locations in a decreasing number of frequencies: hypopharynx (13.3%), nasopharynx (10.0%), oropharynx (10.0%), lung (10.0%), larynx (3.3%) and parotid gland (3.3%). All tonsil carcinomas were ipsilateral to the affected neck site and mostly metastasized into the LN of level II (77.8%). Seven out of 17 endoscopically detected primary tumors were hidden in the tongue base. Furthermore, panendoscopy revealed the hypopharynx (n = 4), oropharynx (n = 3), nasopharynx (n = 2) and larynx (n = 1) as primary site (Table 3).

Within the entire patient cohort (groups A and B), invasive diagnostic methods (panendoscopy or tonsillectomy) led to a higher detection rate than imaging modalities (CT, FDG-PET-CT or MRI). The results showed detection rates of 15.1% and 7.8% for invasive methods and imaging diagnosis, respectively. Tonsillectomy and panendoscopy revealed the primary tumor in 14.9% and 15.2% of patients, whereas the detection rates of CT, MRI and FDG-PET-CT were 10.1%, 4.8% and 6.5%, respectively (Table 3).

In eight patients (10.5%) of group A, the primary tumor emerged during the routine follow-up by performing a CT, MRI or FDG-PET-CT scan, whereas the primary tumor could be found in three patients (3.9%) after autopsy. FDG-PET-CT led to a detection rate of 16.1%, while CT and MRI could identify the primary site in 17.3% and 15.4% of patients during the follow-up. All three imaging modalities showed comparable detection rates. The distribution of the localization was as follows: base of the tongue (n = 5), jaw angle, thymus, oropharynx, hypopharynx, tonsils and lung (one each).

### 3.5. Treatment Methods

#### 3.5.1. Group A—CUP

Initially, ND was performed in 86.8% of patients and was mostly either a radical or modified radical ND (cumulatively: 77.4%). In certain cases, such as N1 status, selective ND (22.6%) or only single lymph node excision (13.2%) was applied. Subsequently, all patients were irradiated bilaterally (61.8%) or unilaterally (38.2%) according to curative radiotherapy (RT) protocol. In both bilateral and unilateral RT, the median dose of the affected neck site was 60 Gy, while the unaffected contralateral site was irradiated with a median dose of 53 Gy in bilaterally applied RT. Moreover, 43.4% of patients received chemotherapy. Patients with palliative therapy protocols (n = 18) were not included in this descriptive analysis (Table 4).

#### 3.5.2. Group B—tCUP

Depending on the localization of the primary tumor, surgical tumor resection, RT and/or CTX were performed with curative intention. Tumor resection included laser resection of tongue base (37.5%) or oropharynx (6.3%), transpalatinal-endonasal tumor resection (6.3%), parotidectomy (6.3%) and tonsillectomy (43.6%). In the case of tonsil cancers, the primary tumor was already removed as part of the diagnostic workup (tonsillectomy). R0 resection could be achieved in 68.8% of cases, whereas a re-resection had to be performed in 13.3%. Within group B, 83.3% of patients received a curative planned RT with both a median dose of 60 Gy for the primary tumor and LN. In one patient (4.0%), only the primary tumor was irradiated. CTX was applied in half of the patients with a median amount of three cycles.

Single LNs were excised in 73.3% of cases, subsequently followed by ND in 60.0% of patients, whereby lymph nodes of level I–V were removed in 49.9% of cases (RND: 16.6%, MRND: 33.3%) (Table 4).

### 3.6. Clinical Outcome

Within the entire patient cohort, the median follow-up time was 38 months (mean: 53.3 months), and an estimated 5-year overall survival (OS) and regional recurrence-free survival (RRFS) of 61.0% and 68.7% were observed, respectively.

Within the follow-up period of 5 years, the median time to death was 16.5 months (mean: 22.68 months) while regional recurrence (RR) occurred after a median time of 10 months (mean: 15.72 months). In 13.2% of patients, distant metastases emerged during the follow-up (Table 4).

#### 3.6.1. Group A—CUP

Within group A, the median follow-up time was 41.00 months (mean: 54.1 months). The overall survival rate was 68.4% with a median time to death of 29 months (mean: 40.5 months) whereas regional recurrences (RR) occurred in 26.3% of patients with a median relapse time of 10 months (mean: 21.5 months).

By using the Kaplan–Meier estimator, a 5-year OS and RRFS of 67.9% and 71.5% was observed, respectively. The median time to death and RR were 25 and 9 months within the observation time of 5 years, respectively (Table 5).

During the follow-up, distant metastases emerged in 6.6% of patients and were localized in the bones (n = 2), lung (n = 1) and liver (n = 1). Furthermore, generalized metastases were observed in two patients.

#### 3.6.2. Group B—tCUP

In group B, the median follow-up time was 21 months (mean: 51.1 months). The overall survival rate was 43.3%, with a median time to death of 12 months (mean: 23.0 months). The occurrence of RR was observed in 36.8% of patients with a median relapse time of 15 months (mean: 17.4 months).

Kaplan–Meier survival analysis led to an estimated 5-year OS and RRFS of 42.6% and 58.2%, respectively. Within the observation period of 5 years, the median time to death was 11 months (mean: 15.6 months), whereas the median relapse time was 15 months (mean: 17.4 months) (Table 5).

The occurrence of distant metastases was observed in 30.0% of patients. Besides generalized distant metastases in five patients, further localizations were in the brain (n = 2), lung (n = 1) and bone (n = 1).

### 3.7. Survival Analyses—Kaplan–Meier and Cox Regression Analyses

#### 3.7.1. Group A vs. B

By performing the log-rank test, the comparison between groups A and B resulted in being significant (*p* = 0.002) regarding 5-year OS. Hence, patients with detected primary tumor during the diagnostic workup (group B) had a significantly worse 5-year OS (42.6% vs. 67.9%), and the median time to death was 11 months versus 25 months in group A (Figure 1a). However, the analysis of the 5-year RRFS did not lead to statistically relevant differences in both groups (*p* = 0.324, Figure 1b). (Table 5).

The univariate Cox regression analysis showed the significantly deteriorating prognostic impact of occurred primary tumors (group B) on the OS (HR: 2.764, *p* = 0.003). Within group A, the occurrence of primary tumors during the follow-up substantially worsened the 5-year OS and RRFS (*p* = 0.007, *p* = 0.027) and further had an impairing prognostic impact on the OS (*p* = 0.012) (Table 5).

Furthermore, patients in group B had a significantly higher probability of emerging distant metastases compared to group A (*p* = 0.001, 30.0% vs. 6.6%).

#### 3.7.2. Impact of Further Clinical Variables on 5-Year OS and RRFS

Advanced nodal disease (N2b-3, *p* = 0.005, *p* = 0.010, *p* = 0.010), the occurrence of RR (*p* = 0.001, *p* = 0.002) and DM (*p* < 0.001, *p* = 0.001, *p* < 0.001) led to both a significant worse 5-year OS and RRFS, respectively, and could also be confirmed as substantially deteriorating prognosticators regarding OS by performing univariate cox regression analyses. Furthermore, significance could be observed for advanced nodal disease (*p* = 0.039) and the emergence of DM (*p* = 0.005) in multivariate cox regression analysis (Table 5).

p16 overexpression had a significantly improving impact on the 5-year RRFS (*p* = 0.013). Furthermore, within group B, advanced tumor progression (>T2) adversely affected the 5-year OS (*p* = 0.005) and RRFS (*p* = 0.009), respectively, and emerged as worsening prognostic factor regarding OS (*p* = 0.010). Further clinical variables did not result in statistically significance (Table 5).

## 4. Discussion

Carcinoma of unknown primary is a rare disease in which large-scale prospective randomized trials are missing, and as a result, standard guidelines based on clinical trials were not yet established [3,9,16]. Therefore, adequate diagnosis and treatment are mainly based on clinicians’ experience and observation. In times of constant imaging and immunohistochemical innovations and novelties, the major challenge is to find the balance between extensive and efficient diagnostic workup without potentially delaying the therapy initiation [17].

In the literature, conventional imaging methods (CT and MRI) were compared to FDG-PET-CT regarding primary tumor detection. CT/MRI could locate the primary site in 9–36% of cases, whereas two meta-analyses described pooled detection rates of 25% and 37% for FDG-PET-CT, respectively [1,6,10,11]. In our study, CT, MRI and FDG-PET-CT could reveal the primary tumor in 10.1%, 4.8% and 6.5% of cases, respectively. Hence, the majority of primary tumors also remained occult after imaging diagnosis in patients with inconspicuous clinical examination. Compared to the literature, the lower detection rates might be due to our restrictive inclusion criteria, as only patients with perfectly unremarkable clinical examination and, therefore, no evidence of the primary site was included. Furthermore, the primary detection rate of FDG-PET-CT during the follow-up was comparable to CT/MRI and did not show a beneficial diagnostic value. This is of utmost importance since the waiting time is prolonged and resources in general regarding PET-CT scans are scarce in some countries. The knowledge about comparable detections rates between these imaging techniques could lead to a shorter detection time and subsequently to better overall survival.

Besides clinical examination and imaging modalities, a further standard part of the diagnosis procedure is the invasive diagnostic part, commonly consisting of tonsillectomy and mandatory panendoscopy. Tonsillectomy is described to yield a primary detection rate ranging from 18 to 41% [14,18,19]. While the authors basically recommend the performance of tonsillectomy, there is disagreement on the extent of tonsillectomy. Generally, carcinomas of the tonsil appear ipsilateral to the presenting neck mass [1,19]; however, the contralateral or bilateral occurrence was also described by several research groups [4,20]. In our study, tonsillectomy revealed the primary tumor in 14.9%, and all were solely present on the affected neck site. Biopsies, which were taken during panendoscopy under general anesthesia, yield the location of the primary site in 24–29% of patients [5,21]. In our patient cohort, panendoscopy led to a primary identification rate of 15.2%, which were most commonly located in the tongue base (41.2%) and hypopharynx (23.5%).

In our study, we could emphasize the importance of thorough clinical examinations, including flexible nasopharyngoscopy in combination with the physician’s experience, as the primary tumor still remained unknown in 75.8% of patients after further extensive diagnosis. Consequently, the question arises, how much time and effort should be put into more extensive diagnosis in addition to conventional methods. Clinicians should consider that extensive diagnostic methods such as FDG-PET-CT can significantly delay therapy initiation due to limited availability, whereas the additional diagnostic value has not yet been proven substantially [4,5,13]. Even though our patients were not randomized due to the retrospective analysis, we could observe a clearly beneficial tendency of invasive diagnostics compared to imaging modalities in detecting the primary site. In particular, the added value of FDG-PET-CT appeared to be debatable with consideration of limited availability, potential therapy delay and comparable detection rates to CT/MRI. Supported by the literature [1], we, therefore, recommend the performance of conventional diagnostic methods comprising thorough clinical examination, including flexible nasopharyngoscopy, ultrasound of the neck, FNAC or ultrasound-guided core biopsy, CT/MRI, tonsillectomy and panendoscopy under general anesthesia. More extensive diagnosis modalities such as FDG-PET-CT should be evaluated individually and only be performed without delaying the start of therapy.

Recently, Herruer et al. presented a new approach to diagnosis and treatment. The authors compared PET-CT findings with intraoperative identification of primary tumors by performing oropharyngeal transoral laser microsurgery. PET-CT led to a true localization of the primary site in 46%. PET-CT failed to detect 32% of the primary site, whereas 12% of the findings were identified false positively, and 10% were true CUPs. In contrast, the primary tumor could be determined intraoperatively on frozen sections and on final histopathology in 82% and 90%, respectively, and achieved histopathologically negative margins in 90%. Followed by neck dissection, the authors conclude the avoidance of adjuvant therapy in one-third of the patients [22]. This novel approach seems to be promising and revolutionizing but needs to be proven in further studies and the diagnostic value and long-term outcome compared to conventional diagnosis and treatment procedures.

The value and benefit of primary site diagnosis are still the subjects of controversy. With certainty, identified primary tumors henceforth enable a tumor-specific therapy and potentially avoid unnecessary interventions, whereas the value of the primary tumor as an outcome prognosticator is discussed controversially [12,13,14]. Issing et al. described a significantly worse 3- and 5-year tumor-specific survival in patients with secondarily occurred primary tumor [13], whereas Fernandez et al. did not observe statistical significance despite a decrease in the 5-year survival rate from 31% to 13% when the primary tumor appeared [12]. To our knowledge, we could analyze true CUP vs. tCUP regarding the outcome for the first time. Our study demonstrated a significantly worse 5-year OS (*p* = 0.002) in patients with occurred primary tumors during the imaging and invasive part of the diagnosis. Furthermore, those primary tumors emerged as significantly deteriorating prognostic markers (*p* = 0.003). Interestingly, patients with tCUP also had a substantially higher probability of distant failure than patients with true CUP (*p* = 0.001). Moreover, the occurrence of primary tumors during the follow-up also had a significantly worsening impact on 5-year OS (*p* = 0.007) and RRFS (*p* = 0.027) and was further an important survival prognosticator (*p* = 0.012). By comparing tCUPs versus primary tumors that occurred during the follow-up, we could observe a decrease in 5-year OS and RRFS of 15.9% and 11.4% in patients with later diagnosed primary sites despite mucosal irradiation of all high-risk sites. Although the results did not lead to statistical significance, it is still an interesting finding during the follow-up, as mucosal irradiation should have lessened the probability of tumor emergence. Hence, potential factors leading to poor survival outcomes after the emergence of primary tumors should be a point of interest in future studies.

In the literature, advanced nodal disease [12,16,23,24], tumor grading [12,16,25], extracapsular spread [23,26] and LN localization [12,16,27] were described as key prognostic factors. Within our patient cohort, advanced nodal stage (N2b-3) as deteriorating survival prognosticator (*p* = 0.010) coincided with previous studies. Moreover, RR (*p* = 0.002) and distant metastases (*p* < 0.001) could be determined as significantly worsening prognostic marker.

Our findings should help clinicians in directing future diagnostic strategies and offers clear indications of an efficient diagnostic approach by focusing on conventional diagnostic modalities. However, large-scale randomized studies are necessary to prove our study results and establish an evidential standard diagnostic algorithm. Those studies may also rule out possible bias and study limitations such as differences in quality and procedure of diagnosis and treatment, follow-up care or information bias that are hardly avoidable in a multicenter, retrospective study design.

## 5. Conclusions

Clinical examination and the physician’s experience were the cornerstone of the primary identification as imaging and invasive diagnostic methods additionally revealed the primary site is only about one-fourth of the patient cohort. Clinical examination in combination with tonsillectomy and panendoscopy was superior to imaging modalities in detecting the primary tumor. Therefore, we recommend, in addition to thorough clinical examination including flexible nasopharyngoscopy, the performance of conventional diagnostic procedures consisting of ultrasound of the neck, CT/MRI, FNAC or ultrasound-guided core biopsy, tonsillectomy and panendoscopy. The initiation of the therapy should not be delayed due to the limited availability of more extensive diagnoses such as FDG-PET-CT. Furthermore, we could emphasize the significantly worsening impact of both early and late occurred primary tumors on clinical outcomes and the importance of deteriorating survival prognosticators.

## Figures and Tables

**Table 1:** Patient characteristics and details of diagnostic methods.

	No. of Patients (%)
**Gender**	
Male	98 (79.0%)
Female	26 (21.0%)
**Age (median, years)**	61.00
**Smoking history**	
No	54 (43.5%)
Yes	70 (56.5%)
Cigarettes per day (median)	20.0
**Alcohol consumption**	
No	35 (28.2%)
Yes	89 (71.8%)
**Cancer in family**	
No	80 (64.5%)
Yes	44 (35.5%)
**Imaging diagnostics**	
CT head/neck	99 (82.5%)
CT thorax	81 (69.2%)
CT abdomen	70 (59.8%)
MRI	42 (34.4%)
FDG-PET-CT	62 (50.0%)
**Invasive diagnostics**	
Fine needle aspiration	44 (35.5%)
Panendoscopy	112 (90.3%)
Tonsillectomy	47 (37.9%)
St.p. Tonsillectomy	47 (37.9%)

Abbreviations: CT = computed tomography, FDG-PET-CT = fluorodeoxyglucose positron emission tomography–computed tomography, MRI = magnetic resonance imaging, No. = number, St.p. = Status post.

**Table 2:** Details of lymph node metastases.

	No. of Patients (%)
**Site of lymph node metastases**	
Left	59 (47.6%)
Right	56 (45.2%)
Bilateral	9 (7.3%)
**Level of lymph node metastases**	
Level I	24 (12.9%)
Only Level I	11
Level II	83 (44.6%)
Only Level II	45
Level III	44 (23.7%)
Only Level III	8
Level IV	20 (10.8%)
Only Level IV	5
Level V	12 (6.4%)
Only Level V	5
Level VI	3 (1.6%)
Only Level VI	2
**N-Classification (AJCC)**	
N1	17 (13.7%)
N2a	16 (12.9%)
N2b	5 (44.4%)
N2c	9 (7.3%)
N3	24 (19.4%)
Not stated	3 (2.4%)
**Histology**	
Squamous cell carcinoma	111 (89.5%)
Adeno carcinoma	6 (4.8%)
Neuroendocrine carcinoma	1 (0.8%)
Undifferentiated carcinoma	2 (1.6%)
Not stated	4 (3.2%)
**Grading**	
G1	3 (2.4%)
G1-G2	2 (1.6%)
G2	46 (37.1%)
G2-G3	13 (10.5%)
G3	42 (33.9%)
G4	2 (1.6%)
Not stated	16 (12.9%)
**p16 status**	
positive	25 (54.3%)
negative	21 (45.7%)
Not stated	78 (62.9%)

Abbreviations: AJCC = American Joint Committee on Cancer, No. = number.

**Table 3:** Primary tumor and diagnostic methods.

	No. of Patients (%)
**Primary tumor**	
Group A—CUP	94 (75.8%)
Group B—tCUP	30 (24.2%)
**Primary tumor detection**	
*Diagnostic methods—Group B*	
Tonsillectomy	7 (70.0%)
Panendoscopy	17 (60.7%)
CT	10 (37.0%)
MRI	2 (28.6%)
FDG-PET-CT	4 (40.0%)
*Diagnostic methods—Group A + B*	
Invasive methods	24 (15.1%)
Tonsillectomy	7 (14.9%)
Panendoscopy	17 (15.2%)
Imaging modalities	16 (7.8%)
CT	10 (10.1%)
MRI	2 (4.8%)
FDG-PET-CT	4 (6.5%)
**Primary tumor localization**	
Tonsil	8 (26.7%)
Ipsilateral tonsil	8 (100.0%)
Tongue base	7 (23.3%)
Hypopharynx	4 (13.3%)
Nasopharynx	3 (10.0%)
Oropharynx	3 (10.0%)
Lung	3 (10.0%)
Larynx	1 (3.3%)
Parotid gland	1 (3.3%)
**Tumor classification (AJCC)**	
T1	14 (46.7%)
T2	7 (23.3%)
T3	1 (3.3%)
T4	4 (13.3%)
Tx	1 (3.3%)
Not stated	3 (10.0%)
**Tumor grading**	
G1	0 (0.0%)
G1-G2	1 (3.3%)
G2	16 (53.3%)
G2-G3	1 (3.3%)
G3	7 (23.3%)
G4	1 (3.3%)
Not stated	4 (13.3%)

Abbreviations: AJCC = American Joint Committee on Cancer, CT = computed tomography, CUP = carcinoma of unknown primary, FDG-PET-CT = fluorodeoxyglucose positron emission tomography-computed tomography, MRI = magnetic resonance imaging, No. = number, RT = radiotherapy, St.p. = Status post, tCUP = turned from CUP to primary carcinoma.

**Table 4:** Treatment methods and clinical outcome.

	No. of Patients (%)
**Group A—CUP**	
Single lymph node excision (SLNE)	38 (50.0%)
Single lymph node excision only	10 (13.2%)
Neck dissection (ND)	66 (86.8%)
Neck dissection only	38 (50.0%)
SLNE + ND	28 (36.8%)
Radiotherapy (RT)	76 (100.0%)
Unilateral	29 (38.2%)
Bilateral	47 (61.8%)
Chemotherapy (CTX)	33 (43.4%)
**Group B—tCUP**	
*Primary tumor*	
Tumor resection	16 (53.3%)
R0	11 (68.8%)
R1	3 (18.8%)
Not stated	2 (12.5%)
Re-Resection	4 (13.3%)
Radiotherapy	25 (83.3%)
Primary tumor only	1 (4.0%)
Chemotherapy	15 (50.0%)
Tumor resection only	3 (10.0%)
Radiotherapy only	4 (13.3%)
Chemotherapy only	1 (3.3%)
Tumor resection + RT	8 (26.7%)
Tumor resection + CTX	1 (3.3%)
RT + CTX	9 (30.0%)
Tumor resection + RT + CTX	4 (13.3%)
*Lymph node metastases*	
Single lymph node excision (SLNE)	22 (73.3%)
SLNE only	10 (33.3%)
Neck dissection (ND)	18 (60.0%)
ND only	6 (20.0%)
SLNE + ND	12 (40.0%)
Radiotherapy (RT)	24 (80.0%)
Unilateral	3 (12.5%)
Bilateral	21 (87.5%)
Chemotherapy (CTX)	15 (50.0%)
**Clinical outcome**	
5-year OS ^1^	61.0%
Time to death (median, months)	16.50
5-year RRFS ^1^	68.7%
Relapse time (median, months)	10.00
Distant metastasis	14 (13.2%)
Follow-up time (median, months)	38.00

Abbreviations: CUP = carcinoma of unknown primary, OS = overall survival, RRFS = regional recurrence-free survival, tCUP = turned from CUP to primary carcinoma. ^1^ Kaplan–Meier Estimator.

**Table 5:** Kaplan–Meier and Cox regression analyses in investigated patients.

		Kaplan–Meier Analyses				Cox Regression Analyses (OS)				
		%		%		Univariate			Multivariate	
	n	5-Year OS	*p* Val. ^1^	5-Year RRFS	*p* Val. ^1^	HR	*p* Val. ^2^	95% CI	HR	*p* Val. ^3^
**Group A vs. B**			0.002		0.324	2.764	0.003	1.403–5.446	1.751	0.135
Group A	76	67.9		71.5						
Group B	30	42.6		58.2						
**PrimFU**			0.007		0.027	3.468	0.012	1.311–9.171	---	---
No	65	74.9		75.5						
Yes	11	26.7		46.8						
**Group B vs. PrimFU**			0.777		0.371	0.872	0.778	0.337–2.257	---	---
Group B	30	42.6		58.2						
PrimFU	11	26.7		46.8						
**Lymph node**										
**N1–2a vs. N2b-3**			0.005		0.010	3.926	0.010	1.379–11.178	3.065	0.039
N1-2a	31	83.2		89.5						
N2b-3	73	52.4		58.5						
**G1-2 vs. >G2**			0.325		0.703	0.705	0.331	0.348–1.426	---	---
G1-2	42	55.0		67.7						
>G2	52	65.2		74.5						
**p16 status**			0.201		0.013	0.482	0.213	0.153–1.522	---	---
positive	30	76.3		84.2						
negative	21	57.9		56.3						
**RR (no vs. yes)**			0.001		---	3.502	0.002	1.584–7.740	---	---
No	68	79.8		---						
Yes	27	40.4		---						
**DM (no vs. yes)**			0.000		0.001	4.972	0.000	2.448–10.118	3.067	0.005
No	92	70.9		74.2						
Yes	14	8.6		18.8						
**Group B**										
**T1-2 vs. >T2**			0.005		0.009	5.053	0.010	1.467–17.400	---	---
T1-2	20	63.7		70.7						
>T2	6	16.7		0.0						
**Tumor resection**			0.635		0.115	0.783	0.637	0.282–2.168	---	---
No	14	40.0		85.7						
Yes	16	46.4		40.0						

Abbreviations: CI = confidence interval, DM = distant metastasis, HR = hazard ratio, OS = overall survival, PrimFU = primary tumor during follow-up, RR = regional recurrence, RRFS = regional recurrence-free survival, val. = value. ^1^ Kaplan–Meier analyses, ^2^ Univariate Cox regression analyses, ^3^ Multivariate Cox regression analyses.
